# The association between social activity and physical frailty among community-dwelling older adults in Japan

**DOI:** 10.1186/s12877-022-03563-w

**Published:** 2022-11-16

**Authors:** Osamu Katayama, Sangyoon Lee, Seongryu Bae, Keitaro Makino, Ippei Chiba, Kenji Harada, Yohei Shinkai, Hiroyuki Shimada

**Affiliations:** 1 Department of Preventive Gerontology, Center for Gerontology and Social Science, National Center for Geriatrics and Gerontology, 7-430 Morioka-cho, Obu City, Aichi 474-8511 Japan; 2 Japan Society for the Promotion of Science, Tokyo, Japan; 3 Department of Health Care and Science, Dong-A University, Busan, South Korea; 4 Tohoku Medical Megabank Organization (ToMMo), Tohoku University, Sendai, Japan

**Keywords:** Social activities, Social participation, Social engagement, Physical frailty, Older adults, Japan

## Abstract

**Background:**

Physical frailty is associated with social activity. However, the relationship between physical frailty and levels of engagement with other people during social activities remains unclear. Thus, we aimed to clarify the relationship between physical frailty and social activity using a taxonomy of activity levels among community-dwelling older adults in Japan.

**Methods:**

This cross-sectional observational study analyzed data from 12,788 older adults (7001 women, mean age: 73.8 years, standard deviation = 5.9; range: 60–96 years) from the National Center for Geriatrics and Gerontology-Study of Geriatric Syndromes. Physical frailty was assessed using the following components: slow walking speed, muscle weakness, exhaustion, low activity, and weight loss. We asked participants about seven social activities that included social participation and engagement and examined their relationship to physical frailty.

**Results:**

Physical frailty was independently associated with all social activities. Exercise circle activity, which includes a level of social participation, was strongly associated with physical pre-frailty and physical frailty. Results of sub-analyses indicated that the level of social engagement was independently associated with physical frailty in the older group (over 75 years) but not in the younger group (60–74 years).

**Conclusions:**

Our results indicate that the strength of the association between social activity and physical frailty differs by the level of social participation. Given the increasingly high prevalence of physical frailty in Japan and its strong association with numerous adverse health outcomes, the relationship between physical frailty and levels of social participation may assist in developing measures to prevent the incidence and progression of physical frailty.

**Supplementary Information:**

The online version contains supplementary material available at 10.1186/s12877-022-03563-w.

## Background

Many developed countries have rapidly aging populations, and Japan’s population has been aging the fastest. By 2020, the number of people aged 65 years and above in Japan had reached 35.9 million—28.4% of the population and the highest proportion globally [1]. Japan is predicted to maintain its position as the country with the oldest population [1]. Frailty in older adults, often defined as a physiological decline in later life [2, 3], is gaining international attention as population aging increases globally [4]. Frailty can lead to several adverse health outcomes [4], such as disability [5], falls [6], hospitalization [7], and mortality [8].

Increasingly, attention is being paid to social activity in later life. Social activities are characterized by interactions with the environment and ingroup members; notably, they bring people together around practices with shared meaning and often engage both the mind and body [9]. The Government of Japan has indicated that, given that Japan is an aging society with diverse values, it will promote and support participation in social activities that enrich spirit and offer a sense of purpose in life [10]. Social participation is a broad concept that can take many forms, including leisure activities, meeting friends, and volunteering [11]. Social participation can be thought of as an individual’s various levels of involvement with others in activities. These levels of involvement can be presented as a hierarchy, which may be used to distinguish social participation from similar concepts, such as social engagement [11]. A systematic review by Levasseur and colleagues proposed six levels of social activities (a taxonomy of social participation). Notably, doing activities alone (Level 1) or in parallel (Level 2) is not considered social participation. The other levels include interaction with others and are regarded as social participation. More specifically, Level 3 concerns socially-oriented activities (e.g., talking with neighbors) and Level 4 involves task-oriented activities (e.g., computer classes at a senior center). Level 5 activities are oriented toward helping others (e.g., volunteering) and Level 6 activities are society-oriented (e.g., being involved in a political party).

Physical frailty and social participation have been associated with reduced frequency of social participation [12] and engagement in activities with others [13, 14]. However, few reports have categorized social activity at the level of engagement with others and examined its relation to physical frailty, and their results are not sufficiently clear.

Understanding the relationship between physical frailty and levels of social participation may be helpful for developing measures to prevent the incidence and progression of physical frailty. Therefore, the purpose of this study was to clarify the relationship between physical frailty and social activity among community-dwelling older adults in Japan using the taxonomy of activity levels proposed by Levasseur and colleagues [11]. It was hypothesized that the strength of the association between physical frailty and social activity would differ with the level of social activity.

## Methods

### Participants

The data used in this study were obtained from the National Center for Geriatrics and Gerontology-Study of Geriatric Syndromes (NCGG-SGS) [15], a study on health promotion for older adults in the Midori Ward of Nagoya, Tokai, and Takahama in Japan. Our inclusion criteria were as follows: all participants had to reside in the Midori Ward of Nagoya, Tokai, or Takahama and be at least 70 years or older in the Midori Ward of Nagoya, 65 years or older in Tokai, and 60 years or older in Takahama at the time of the study. Takahama’s age was set at 60 years or older because many people in Japan reach retirement at 60 years of age, and the risk of health problems, such as frailty and disability, is thought to increase due to major lifestyle changes during this time. A total of 14,987 community-dwelling older adults participated in face-to-face interviews and physical and cognitive function assessments. The exclusion criteria were: (1) the need for support/care, as certified by the Japanese public long-term care insurance system—due to disability (*n* = 158); (2) having a disability that affects basic activities of daily living (ADL; *n* = 25); (3) having health problems (i.e., dementia, stroke, or Parkinson’s disease) *n* = 975; and (4) responses with missing measurement variables (*n* = 1041). Health problems were interviewed face-to-face by our trained and qualified nurses from the participants.

**Fig. 1:**
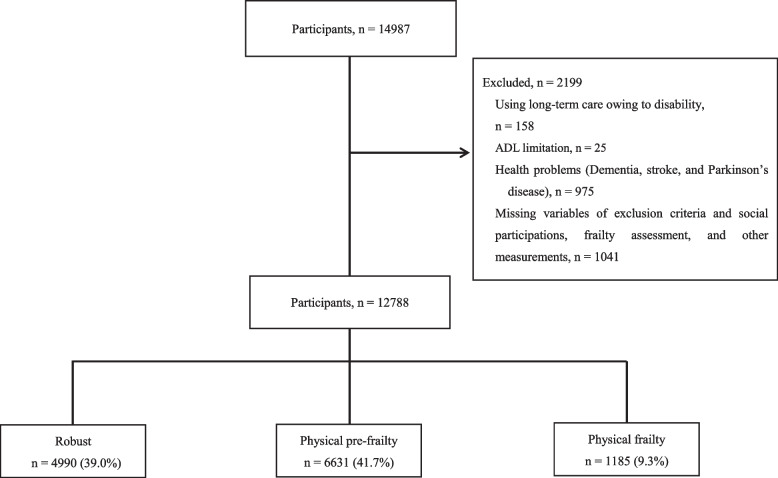
Flow diagram of sample selection

Of the initial 14,987 participants, 2199 were excluded; thus, the final analysis included data from 12,788 older adults (7001 women; mean age: 73.8 years, standard deviation [SD] = 5.9; age range: 60–96 years) (Fig. 1). The study was conducted according to the Declaration of Helsinki. All participants provided written informed consent before being included in the study. The study protocol was approved by the Ethics Committee of the National Center for Geriatrics and Gerontology (No. 1440–3).

### Measurement of social activities

Social participation was assessed using results from a self-report questionnaire available in the NCGG-SGS dataset [16], the Japan Science and Technology Agency (JST-IC) [17], and the Kihon Checklist (KCL) [18]. Data regarding participation in seven social activities were collected depending on participants’ answers to the following questions: (1) “Do you sometimes visit your friends?” (Visit friends), (2) “Do you go shopping to buy daily necessities by yourself?” (Shopping), (3) “Do you go to a group exercise circle?” (Exercise circle), (4) “Do you cooperate in regional events (e.g., assisting in organizing events, making flyers, and organizing festivals)?” (Cooperation in regional events), (5) “Do you turn to your family or friends for advice?” (Advice), (6) “Do you engage in any activities related to environmental beautification (e.g., cleaning up parks)?” (Voluntary activities), and (7) “Are you a board member or a secretary of a neighborhood association, senior citizens’ club, or nonprofit organization?” (Board member or secretary).

In this study, questions (1–2) corresponded to Level 3, (3–4) to Level 4, (5) to Level 5, and (6–7) to Level 6. Furthermore, following the previous study, Levels 3 and 4 were categorized as social participation, while Levels 5 and 6 were categorized as social engagement [11].

Participants were asked to respond with “yes” or “no” to the social participation questions based on their activities within the past month and to the social engagement questions based on their involvement in activities during the past year. The social activities were then divided into four categories: “Both active,” “Social engagement only active,” “Social participation only active,” or “Both inactive.” In this study, we divided them by the receiver operating characteristic curve, area under the curve, and Youden index (Supplementary Materials).

### Physical frailty assessment

Following Fried and colleagues’ original study, this study considered physical frailty as satisfying three of the following criteria [2]: slow walking speed, weakness, exhaustion, low physical activity, and weight loss. Participants showing none of these components were considered to be robust, those showing one or two components were considered to be pre-frail, and those showing three or more components were considered to be frail. Walking speed was measured using a detailed protocol described in a previous study [19]. The participants walked on a flat and straight surface at a comfortable speed, and markers were used to indicate both the start and the end of a 2.4 m walking path. A 2 m section was marked at the start and end of the path. Patients traversed this section before passing the start marker, so that they were walking at a comfortable pace when they reached the timed path. To ensure a consistent walking pace while on the timed path, participants were asked to continue walking for an additional 2 m past the end of the timed path. Using the cut-off of Fried and colleagues’ Cardiovascular Health Study (CHS) criteria may have caused the Japanese to overlook frailty; unified CHS frailty index criteria that were more suited to Japanese older adults were required [20]. Therefore, in a study conducted with Japanese older adults, a cut-off for walking speed of 1.0 m/s or higher was shown to distinguish between independent, healthy older adults and those who need support in daily living [21]. Additionally, the Asian Working Group for Sarcopenia 2019 used 1.0 m/s as a cut-off [22]. Accordingly, in this study, 1.0 m/s was established as slow walking speed [21, 23].

Weakness, measured in kilograms, was defined according to maximum grip strength using a Smedley-type handheld dynamometer (GRIP-D; Takei Ltd., Niigata, Japan). Sex-specific cutoffs (< 26 kg for men and < 18 kg for women) were used to establish weakness [24]. If the participant responded “yes” to the question, “In the last two weeks, have you felt tired without a reason?” they were considered exhausted. The question about feeling tired was taken from the KCL, a comprehensive self-report checklist of health items developed by the Japanese Ministry of Health, Labour and Welfare [25]. We evaluated physical activity using the following questions about time spent engaged in sports and exercise: “Do you engage in moderate levels of physical exercise or sports aimed at health?” and “Do you engage in low levels of physical exercise aimed at health?” [23]. If participants answered “no” to both of these questions, we considered them to engage in low levels of physical activity [26]. Weight loss was assessed by a response of “yes” to the question “Have you lost 2 kg or more in the past six months?” [25].

### Potential confounding factors

Factors such as demographic variables, chronic diseases, psychological factors, and metabolic parameters associated with frailty and social participation in older adults could be potentially confounding [4, 27, 28]. Therefore, our model included the following covariates: age at enrollment, sex, years of education, medications, chronic diseases (i.e., heart disease, hypertension, diabetes, and hyperlipidemia), Mini-Mental State Examination (MMSE) score [29], self-rated health (SRH) [30], 15-item Geriatric Depression Scale (GDS) score [31], Body Mass Index (BMI), total body fat, Appendicular Skeletal Muscle Mass (ASM), and frequency of going out in a week. The following self-reported chronic diseases were also included: heart disease, hypertension, diabetes, and hyperlipidemia.

SRH was measured using a single question, “In general, how would you rate your health?” with the following response alternatives: good, rather good, poor, and very poor [30]. The responses were combined into two categories: good (good and rather good) and poor (poor and very poor) [32]. Total body fat and ASM were measured with a bioelectrical impedance analyzer, Tanita MC780A (Tanita Corporation, Tokyo, Japan). This analyzer was developed to estimate body fat based on the principle of bioelectrical impedance analysis [33]. The ASM was derived as the sum of fat-free soft tissues in the arms and legs, assuming that all non-fat and non-bone tissue was skeletal muscle. Frequency of going out in a week was measured by using a Life-Space Assessment (LSA) [34] item. In this study, seven possible responses, from 0 to 6, to indicate the number of days as a measure of a participant’s frequency of going out were replaced with the choice of “no” or “yes” to the question of “going out daily” as a categorical variable.

### Data analysis

We used the Kolmogorov-Smirnov test to confirm the normality of the data. For non-parametric scores that were non-normally distributed, we used the Kruskal–Wallis test. The Kruskal–Wallis test was followed by analysis using the Bonferroni correction for the Mann-Whitney U-test to identify different characteristics among the robust, physical pre-frailty, and physical frailty groups. The categorical variables were compared using Pearson’s chi-squared test. Residuals followed the *t* distribution, wherein *t* > 1.96 indicated *p* < .05. Multinomial logistic regression analysis was used to examine the association of social participation, and frailty type was set as the dependent variable (with the most typical group, robust, as the reference group) after adjusting for covariates. The adjusted model was modified for age at enrollment, sex, years of education, medications, heart disease, hypertension, diabetes, hyperlipidemia, MMSE score, SRH, GDS score, BMI, total body fat, ASM, and frequency of going out in a week. The multinomial logistic regression was developed with the forced-entry method. Data are presented as odds ratios (ORs) with a 95% confidence interval (CI). As age has a crucial effect on social participation, we conducted sub-analyses by applying the regression models to the young-old (60–74 years) and old-old (over 75 years) groups separately. Statistical significance was set at *p* < .05 in all analyses. All analyses were performed using IBM SPSS version 25.0 (IBM Japan, Tokyo).

## Results

The final analysis included data from 12,788 participants (7001 women; mean age: 73.8 years, SD = 5.9; age range: 60–96 years). The three different groups of robust, physical pre-frailty, and physical frailty accounted for 4990 (39.0%), 6613 (51.7%), and 1185 (9.3%) of the participants, respectively. The possible confounding factors for social participation, grouped according to participants’ frailty status, are shown in Table 1. Significant differences were observed among the three groups regarding age, sex, years of education, medications, heart disease, hypertension, diabetes, hyperlipidemia, walking speed, grip strength, MMSE score, SRH, GDS score, BMI, total body fat, ASM, frequency of going out in a week, and each activity of social participation (all *p* < .05).

**Table 1:** Demographic characteristics of older adults by frailty status

	Total ***n*** = 12,788	Robust ***n*** = 4990 (39.0%)	Physical pre-frailty ***n*** = 6613 (51.7%)	Physical frailty ***n*** = 1185 (9.3%)	***P*** value	Post hoc
**Demographic characteristics**						
Age, year. Median (IQR)	74.0 (70.0–78.0)	73.0 (69.0–76.0)	74.0 (70.0–78.0)	78.0 (73.0–82.0)	<.001^a^	Robust < pre-PF < PF
Sex, female (%)	7001 (54.7)	2695 (54.0)	3616 (54.7)	690 (58.2) ^c^	.03^b^	
Education, year. Median (IQR)	12.0 (9.0–13.0)	12.0 (10.0–13.0)	12.0 (9.0–12.0)	10.0 (9.0–12.0)	<.001^a^	PF < pre-PF < Robust
Medication, number. Median (IQR)	2.0 (1.0–4.0)	2.0 (1.0–4.0)	3.0 (1.0–5.0)	4.0 (2.0–6.0)	<.001^a^	Robust < pre-PF < PF
**Chronic diseases**						
Heart disease, no (%)	10,612 (83.0)	4241 (85.0) ^c^	5476 (82.8)	895 (75.5) ^d^	<.001^b^	
Hypertension, no (%)	6836 (53.5)	2863 (57.4) ^c^	3429 (51.9) ^d^	544 (45.9) ^d^	<.001^b^	
Diabetes, no (%)	11,095 (86.8)	4481 (89.8) ^c^	5665 (85.7) ^d^	949 (80.1) ^d^	<.001^b^	
Hyperlipidemia, no (%)	8081 (63.2)	3221 (64.5) ^c^	4119 (62.3) ^d^	741 (62.5)	.04^b^	
**Physical function**						
Walking speed, m/s. Median (IQR)	1.1 (1.0–1.3)	1.2 (1.1–1.3)	1.1 (.9–1.2)	.9 (.8–1.0)	<.001^a^	PF < pre-PF < Robust
Grip strength, kg. Median (IQR)	25.5 (21.1–32.8)	27.6 (22.7–34.9)	25.0 (20.6–32.0)	20.4 (16.4–25.4)	<.001^a^	PF < pre-PF < Robust
**Cognitive function**						
MMSE, score. Median (IQR)	27.0 (25.0–29.0)	28.0 (26.0–29.0)	27.0 (25.0–29.0)	26.0 (24.0–28.0)	<.001^a^	PF < pre-PF < Robust
SRH, good (%)	11,044 (86.4)	4680 (93.8) ^c^	5584 (84.4) ^d^	780 (65.8) ^d^	<.001^b^	
GDS, score. Median (IQR)	2.0 (1.0–4.0)	1.0 (.0–3.0)	2.0 (1.0–4.0)	4.0 (2.0–7.0)	<.001^a^	Robust < pre-PF < PF
**Metabolic parameters**						
BMI, Median (IQR)	23.0 (21.1–25.1)	22.9 (21.1–24.8)	23.1 (21.1–25.3)	23.0 (20.6–25.4)	<.001^a^	Robust < pre-PF
Total body fat, %. Median (IQR)	27.7 (22.3–33.7)	26.8 (21.6–32.5)	28.1 (22.6–34.2)	30.0 (23.9–36.0)	<.001^a^	Robust < pre-PF < PF
ASM, kg. Median (IQR)	16.0 (13.7–20.1)	16.4 (14.0–20.6)	16.0 (13.7–20.0)	15.0 (12.8–18.8)	<.001^a^	PF < pre-PF < Robust
Frequency of going out in a week, yes (%)	6919 (54.1)	2862 (57.4) ^c^	3531 (53.4)	526 (44.4) ^d^	<.001^b^	
**Social participation**						
**Level 3**						
Visit friends, yes (%)	9914 (77.5)	4093 (82.0) ^c^	5020 (75.9) ^d^	801 (67.6) ^d^	<.001^b^	
Shopping, yes (%)	12,278 (96.0)	4842 (97.0) ^c^	6341 (95.9)	1095 (92.4) ^d^	<.001^b^	
**Level 4**						
Exercise circle, yes (%)	3560 (27.8)	1866 (37.4) ^c^	1525 (23.1) ^d^	169 (14.3) ^d^	<.001^b^	
Cooperate in regional events, yes (%)	4559 (35.7)	2025 (40.6) ^c^	2216 (33.5) ^d^	318 (26.8) ^d^	<.001^b^	
**Social engagement**						
**Level 5**						
Advice, yes (%)	11,456 (89.6)	4660 (93.4) ^c^	5860 (88.6) ^d^	936 (79.0) ^d^	<.001^b^	
**Level 6**						
Voluntary activities, yes (%)	4058 (31.7)	1798 (36.0) ^c^	1974 (29.9) ^d^	286 (24.1) ^d^	<.001^b^	
Board member or secretary, yes (%)	3749 (29.3)	1694 (33.9) ^c^	1813 (27.4) ^d^	242 (20.4) ^d^	<.001^b^	

*Abbreviations: IQR* Interquartile range, *pre-PF* Physical pre-frailty, *PF* Physical frailty, *MMSE* Mini-Mental State Examination, *SRH* self-rated health, *GDS* 15-item Geriatric Depression Scale, *BMI* Body Mass Index, *ASM* Appendicular Skeletal Muscle Mass

^a^*p-*Values reported from Kruskal−Wallis test, the Bonferroni correction for the Mann−Whitney U-test

^b^*p-*Values obtained by Pearson’s chi-square test

^c^ Statistically significant association by adjusted standardized residual > 1.96 (*p* < .05)

^d^ Statistically significant association by adjusted standardized residual <−1.96 (*p* < .05)

Table 2 shows the ORs and 95% CIs estimated by both unadjusted and adjusted multinomial logistic regression analyses, with frailty status as the dependent variable (with the robust group as reference) and the element of social activities and each social activity group as the independent variables. After adjusting for potential confounding factors (i.e., demographic variables, chronic diseases, psychological factors, and metabolic parameters), the physical pre-frailty group was found to be independently associated with all items except shopping and social engagement (all *p* < .05). The physical frailty group was independently associated with all items (all *p* < .05). Among the social activities that included social participation, the “exercise circle” activity was the most highly associated with physical pre-frailty and physical frailty groups. In the sub-analyses of the young-old and old-old groups (Table 3 and Table 4), “visit friend,” “exercise circle,” “cooperation in regional events,” “advice,” and “social participation only” (both active and inactive) were all significantly related to physical frailty in the young-old group (all *p* < .05). Meanwhile, all social activity variables were significantly related to physical frailty in the old-old group (i.e., Level 6 of social engagement was independently associated with physical frailty in the older group) (all *p* < .05).

**Table 2:** Multinomial logistic regression analysis with frailty status as the dependent variable

	Unadjusted model						Adjusted model					
	Pre-frailty			Frailty			Pre-frailty			Frailty		
	OR	95% CI	*P* value	OR	95% CI	*P* value	OR	95% CI	*P* value	OR	95% CI	*P* value
**Social participation**												
**Level 3**												
Visit friends, no	1.45	1.32, 1.59	<.001	2.19	1.90, 2.52	<.001	1.26	1.14, 1.39	<.001	1.41	1.20, 1.67	<.001
Shopping, no	1.40	1.15, 1.72	<.001	2.69	2.05, 3.52	<.001	1.18	0.95, 1.47	.13	1.55	1.12, 2.14	<.001
**Level 4**												
Exercise circle, no	1.99	1.84, 2.16	<.001	3.59	3.02 , 4.27	<.001	1.86	1.71, 2.03	<.001	2.84	2.35 3.42	<.001
Cooperate in regional events, no	1.36	1.26, 1.46	<.001	1.86	1.62, 2.14	<.001	1.23	1.14, 1.34	<.001	1.39	1.19, 1.63	<.001
**Social engagement**												
**Level 5**												
Advice, no	1.82	1.59, 2.08	<.001	3.76	3.14, 4.49	<.001	1.28	1.11, 1.48	<.001	1.62	1.31, 1.99	<.001
**Level 6**												
Voluntary activities, no	1.32	1.22, 1.43	<.001	1.77	1.53, 2.05	<.001	1.21	1.11, 1.32	<.001	1.33	1.13, 1.56	<.001
Board member or secretary, no	1.36	1.26, 1.47	<.001	2.00	1.72, 2.33	<.001	1.18	1.08, 1.28	<.001	1.31	1.10, 1.55	<.001
Both inactive, yes	2.14	1.89, 2.42	<.001	4.46	3.69, 5.38	<.001	1.69	1.48, 1.93	<.001	2.18	1.75, 2.71	<.001
Social engagement only active, yes	1.42	1.12, 1.82	<.001	2.48	1.69, 3.63	<.001	1.29	1.00, 1.67	.05	1.79	1.16, 2.77	<.001
Social participation only active, yes	1.31	1.21, 1.41	<.001	1.82	1.56, 2.12	<.001	1.17	1.08, 1.28	<.001	1.34	1.13, 1.58	<.001
Both active, yes	1.00			1.00			1.00			1.00		

The adjusted model is adjusted for age, sex, education, heart disease, hypertension, diabetes, hyperlipidemia, medication, self-rated health, GDS, BMI, Total body fat, ASM, MMSE, and LSA

*Abbreviations: CI* confidence interval, *OR* odds ratio, *GDS* 15-item Geriatric Depression Scale, *BMI* Body Mass Index, *ASM* Appendicular Skeletal Muscle Mass, *MMSE* Mini-Mental State Examination, *LSA* Life-Space Assessment

**Table 3:** Demographic characteristics of older adults by age status

	Young-old ***n*** = 7266 (56.8%)	Old-old ***n*** = 5522 (43.2%)	***P*** value	Post hoc
**Demographic characteristics**				
Age, year. Median (IQR)	71.0 (67.0–73.0)	78.0 (76.0–81.0)	<.001^a^	Young-old < Old-old
Sex, Female (%)	4036 (57.6) ^c^	2965 (42.4) ^d^	.04^b^	
Education, year. Median (IQR)	12.0 (10.0–13.0)	12.0 (9.0–12.0)	<.001^a^	Old-old < Young-old
Medication, number. Median (IQR)	2.0 (1.0–4.0)	3.0 (2.0–5.0)	<.001^a^	Young-old < Old-old
**Chronic diseases**				
Heart disease, no (%)	6257 (59.0) ^c^	4355 (41.0) ^d^	<.001^b^	
Hypertension, no (%)	4346 (63.6) ^c^	2490 (36.4) ^d^	<.001^b^	
Diabetes, no (%)	6330 (57.1)	4765 (42.9)	.17^b^	
Hyperlipidemia, no (%)	4664 (57.7) ^c^	3417 (42.3) ^d^	.01^b^	
**Physical function**				
Walking speed, m/s. Median (IQR)	1.2 (1.0–1.3)	1.1 (.9–1.2)	<.001^a^	Old-old < Young-old
Grip strength, kg. Median (IQR)	26.6 (22.1–34.7)	24.1 (19.9–30.5)	<.001^a^	Old-old < Young-old
**Cognitive function**				
MMSE, score. Median (IQR)	28.0 (26.0–29.0)	26.0 (24.0–28.0)	<.001^a^	Old-old < Young-old
SRH, good (%)	6344 (57.4) ^c^	4700 (42.6) ^d^	<.001^b^	
GDS, score. Median (IQR)	2.0 (1.0–4.0)	2.0 (1.0–4.0)	<.001^a^	Young-old < Old-old
**Metabolic parameters**				
BMI. Median (IQR)	23.1 (21.1–25.1)	22.9 (21.0–25.0)	<.001^a^	Old-old < Young-old
Total body fat, %. Median (IQR)	27.3 (22.1–33.4)	28.2 (22.6–34.0)	<.001^a^	Young-old < Old-old
ASM, kg. Median (IQR)	16.4 (14.0–20.7)	15.6 (13.2–19.5)	<.001^a^	Old-old < Young-old
Frequency of going out in a week, yes (%)	4007 (57.9) ^c^	2912 (42.1) ^d^	.007^b^	
**Social participation**				
**Level 3**				
Visit friends, yes (%)	5603 (56.5)	4311 (43.5)	.199^b^	
Shopping, yes (%)	6985 (56.9)	5293 (43.1)	.423^b^	
**Level 4**				
Exercise circle, yes (%)	1925 (54.1) ^d^	1635 (45.9) ^c^	<.001^b^	
Cooperate in regional events, yes (%)	2434 (53.4) ^d^	2125 (46.6) ^c^	<.001^b^	
**Social engagement**				
**Level 5**				
Advice, yes (%)	6627 (57.8) ^c^	4829 (42.2) ^d^	<.001^b^	
**Level 6**				
Voluntary activities, yes (%)	2186 (53.9) ^d^	1872 (46.1) ^c^	<.001^b^	
Board member or secretariat, yes (%)	2056 (54.8) ^d^	1693 (45.2) ^c^	<.004^b^	
**Frailty status**			<.001^b^	
Robust, yes (%)	3265 (65.4) ^c^	1725 (34.6) ^d^		
Physical pre-frailty, yes (%)	3602 (54.5) ^d^	3011 (45.5) ^c^		
Physical frailty, yes (%)	399 (33.7) ^d^	786 (66.3) ^c^		

*Abbreviations: IQR* Interquartile Range, *MMSE* Mini-Mental State Examination, *SRH* self-rated health, *GDS* 15-item Geriatric Depression Scale, *BMI* Body Mass Index, *ASM* Appendicular Skeletal Muscle Mass

^a^*p*-values reported from Kruskal−Wallis test, the Bonferroni correction for the Mann−Whitney U-test

^b^*p*-values obtained by Pearson’s chi-square test

^c^ Statistically significant association by adjusted standardized residual > 1.96 (*p* < .05)

^d^ Statistically significant association by adjusted standardized residual <−1.96 (*p* < .05)

**Table 4:** Multinomial logistic regression analysis with frailty status as the dependent variable

	Young-old						Old-old					
	Pre-frailty			Frailty			Pre-frailty			Frailty		
	OR	95% CI	*P* value	OR	95% CI	*P* value	OR	95% CI	*P* value	OR	95% CI	*P* value
**Social participation**												
**Level 3**												
Visit friends, no	1.13	1.00, 1.28	.05	1.43	1.11, 1.84	.01	1.35	1.14, 1.60	<.001	1.40	1.11, 1.78	.01
Shopping, no	.88	0.67, 1.15	.35	1.07	.64, 1.81	.79	1.87	1.23, 2.85	<.001	2.38	1.44, 3.92	<.001
**Level 4**												
Exercise circle, no	1.70	1.52, 1.90	<.001	2.67	1.93, 3.69	<.001	1.99	1.74, 2.27	<.001	2.98	2.35, 3.79	<.001
Cooperate in regional events, no	1.12	1.01, 1.25	.04	1.42	1.08, 1.85	.01	1.31	1.15, 1.49	<.001	1.37	1.11, 1.68	<.001
**Social engagement**												
**Level 5**												
Advice, no	1.13	0.94, 1.37	.19	1.89	1.37, 2.61	<.001	1.43	1.14, 1.79	<.001	1.54	1.15, 2.06	<.001
**Level 6**												
Voluntary activities, no	1.11	1.00, 1.24	.06	1.26	.96, 1.64	.10	1.27	1.12, 1.45	<.001	1.34	1.09, 1.66	.01
Board member or secretary, no	1.08	0.97, 1.21	.17	1.33	1.00, 1.76	.05	1.30	1.14, 1.49	<.001	1.34	1.08, 1.68	.01
Both inactive, yes	1.44	1.23, 1.70	<.001	2.12	1.49, 3.00	<.001	1.89	1.49, 2.38	<.001	2.21	1.62, 3.01	<.001
Social engagement only active, yes	.99	0.73, 1.35	.95	1.63	.85, 3.11	.14	1.96	1.20, 3.18	.01	2.35	1.23, 4.49	.01
Social participation only active, yes	1.07	0.96, 1.20	.22	1.43	1.08, 1.90	.01	1.28	1.12, 1.47	<.001	1.32	1.06, 1.64	.01
Both active, yes	1.00			1.00			1.00			1.00		

The adjusted model is adjusted for age, sex, education, heart disease, hypertension, diabetes, hyperlipidemia, medication, self-rated health, GDS, BMI, total body fat, ASM, MMSE, and LSA

*Abbreviations: CI* confidence interval, *OR* odds ratio, *GDS* 15-item Geriatric Depression Scale, *BMI* Body Mass Index, *ASM* Appendicular Skeletal Muscle Mass, *MMSE* Mini-Mental State Examination, *LSA* Life-Space Assessment

## Discussion

The aim of this study was to clarify the association between elements of social activities and physical frailty among community-dwelling older adults in Japan. As initially hypothesized, the strength of the association with physical frailty was revealed to differ by the level of social activity.

The robust, physical pre-frailty, and physical frailty groups accounted for 4990 (39.0%), 6613 (51.7%), and 1185 (9.3%) participants of the total sample, respectively. Physical frailty is present in millions of older adults worldwide. However, the global prevalence of frailty is not yet known, partly because frailty research has predominantly been conducted in high-income countries [4]. In a recent study that used a definition of physical frailty similar to the present study, the prevalence was 8.2% [35], 10.7% [5], and 11.3% [23], respectively. In the current study, comparisons of the three different physical frailty groups showed significant differences between the groups for all items. In particular, the physical frailty group confirmed the general characteristics of demographic factors (older age, more women, and fewer years of education) and clinical factors (chronic diseases, impaired cognition, higher depression score, and obesity) compared to the robust group [4, 36–38]. A similar trend was found in the physical pre-frailty group. Physical frailty is globally considered to be reversible and preventable in its initial phases [39], and interest in physical pre-frailty is increasing [40]. Regarding social participation, there were significantly fewer participants in the physical frailty group than in the robust group on all items. In previous studies, social participation has been found to be limited in older adults with physical frailty [13, 14].

A multinomial logistic regression analysis with frailty type as the dependent variable revealed that social participation and engagement were significantly associated with physical pre-frailty and physical frailty. Among the types of social participation, Level 4 “exercise circle,” and among the social engagement items, Level 5 “advice,” were most strongly associated with physical frailty. These activities can be differentiated by the goals of the activity (Level 4: task-oriented, Level 5: oriented toward helping others) [11]. Lifestyle factors relating to the onset or progression of physical frailty include physical inactivity, and social factors include living alone and loneliness [41]. Level 4, “exercise circle,” was suggested to be highly associated with physical frailty because Level 4 of social participation is defined as, “the individual collaborates with others to perform an activity, reach a common goal,” [11] and includes both lifestyle and social factors of physical frailty. The results of this study were considered to support those of a previous review [41].

Interestingly, this study found that Level 5, “oriented toward helping others,” and Level 6, “society-oriented” activities for others, which are included in social engagement [11], were highly associated with physical pre-frailty and physical frailty. Previous studies have differentiated between two kinds of engagement: social participation and social engagements [11, 42]. While social participation involves less formal engagement with friends and family, social engagement necessarily involves a desire for social change or is considered to impact community choices.

Previous studies on older adults’ volunteerism have shown the beneficial effects of volunteering among older adults, as volunteer work is associated with improved quality of life [43], better psychosocial, physical, and cognitive health, improved life satisfaction, enhanced social support, and delayed mortality [44, 45]. Volunteer work among older people is motivated by the desire to help others, find a peer group, offset losses associated with retirement or a decline in health [46], and give something back to the community and the availability of time [47]. Therefore, we suggest that a desire for helping others may be key to the prevention of physical frailty, which needs to be examined in detail in future studies.

In the young-old group, activities at Levels 3–5 were associated with physical frailty, whereas in the old-old group, all activities at Levels 3–6 were associated with physical frailty. According to a report on the actual situation of social participation of Japanese older adults regarding the status of social activities among those aged 60 and older, 71.9% of those aged 60–69 and 47.5% of those aged 70 and above were either working or engaged in volunteer activities, community activities (e.g., neighborhood associations, community events), hobbies, and other activities [10]. Additionally, the young-old group had a greater frequency of going out in a week than the old-old group, as assessed by the LSA, with more participants indicating that they went out every day. In Japan, the retirement age for employees is required by law to be at least 60. Therefore, the results suggest that the young-old group may have included older adults who were still engaged in work and other social activities, in addition to the social participation measured in this study. Hence, it is possible that the young-old group had only Levels 3–5 social activities associated with physical frailty.

The strengths of this study include its large sample size and assessment to identify physical frailty. To the best of our knowledge, this is the first study to classify levels of an individual’s involvement with others in social activities with different goals and examine the relationship between those levels and physical frailty. However, this study also has some limitations. First, the cross-sectional design requires that the causal relationship between social participation and physical frailty be clarified in future prospective studies. Second, this study did not use random sampling for data collection; hence, the incidence rate of physical frailty among older adults may be under-reported. Finally, this study fails to address other covariates related to biological factors (e.g., cytokines, androgen deficiency, and low carotenoids). These covariates could also affect cumulative age-related changes; therefore, future studies should include these factors. Despite these limitations, this study found that the strength of the association between physical frailty and social activity differs with varying levels of social activity.

## Conclusions

The results of this study indicate that the strength of the association with physical frailty differed by the level of social activity. Given the increasingly high prevalence of physical frailty and its strong association with numerous adverse health outcomes, clinicians can deliver more effective care to older adults by considering their daily levels of social participation, which in turn may lead to better outcomes in the primary prevention of disease.

## Supplementary Information

**Additional file 1: Supplementary Materials.** Receiver operating characteristic curve for social participation and social engagement used to detect the cut point for social inactivity. The Youden index resulted in two “No” cut points for social participation and one “No” cut point for social engagement. AUC, Area Under Curve; 95%CI, 95% confidence interval. Social participation (a), Social engagement (b).

## Data Availability

The datasets used and/or analyzed during the present study are available from the corresponding author on reasonable request.

## Abbreviations

NCGG-SGS: National Center for Geriatrics and Gerontology-Study of Geriatric Syndromes
ADL: activities of daily living
SD: standard deviation
JST-IC: Japan Science and Technology Agency
KCL: Kihon Checklist
MMSE: Mini-Mental State Examination
SRH: Self-rated health
GDS: 15-item Geriatric Depression Scale
BMI: Body Mass Index
ASM: Appendicular skeletal muscle Mass
LSA: Life-Space Assessment
OR: Odds Ratio
CI: Confidence interval
