# Supplementary material for: The association between social activity and physical frailty among community-dwelling older adults in Japan
Source: BMC Geriatr. 2022 Nov 16;22:870. doi: 10.1186/s12877-022-03563-w (PMC9670639; doi:10.1186/s12877-022-03563-w)
Supplement: Supplementary file 1 — Additional file 1: Supplementary Materials. Receiver operating characteristic curve for social participation and social engagement used to detect the cut point for social inactivity. The Youden index resulted in two “No” cut points for social participation and one “No” cut point for social engagement. AUC, Area Under Curve; 95%CI, 95% confidence interval. Social participation (a), Social engagement (b). [file 12877_2022_3563_MOESM1_ESM.docx]

**Supplementary Materials.** Receiver operating characteristic curve for social participation and social engagement used to detect the cut point for social inactivity.

(b)

(a)

The Youden index resulted in two “No” cut points for social participation and one “No” cut point for social engagement.

AUC, Area Under Curve; 95%CI, 95 percent confidence interval. Social participation (a), Social engagement (b).
